# Acid sphingomyelinase deficiency in France: a retrospective survival study

**DOI:** 10.1186/s13023-024-03234-6

**Published:** 2024-08-05

**Authors:** Wladimir Mauhin, Nathalie Guffon, Marie T. Vanier, Roseline Froissart, Aline Cano, Claire Douillard, Christian Lavigne, Bénédicte Héron, Nadia Belmatoug, Yurdagül Uzunhan, Didier Lacombe, Thierry Levade, Aymeric Duvivier, Ruth Pulikottil-Jacob, Fernando Laredo, Samia Pichard, Olivier Lidove, ASSUR Study Group, Marie-Thérèse Abi-Wardé, Marc Berger, Emilie Berthoux, Aurélie Cabannes-Hamy, Fabrice Camou, Pascal Cathebras, Vincent Grobost, Jérémy Keraen, Alice Kuster, Bertrand Lioger, Anas Mehdaoui, Claire Merlot, Martin Michaud, Martine-Louise Reynaud-Gaubert, Fréderic Schlemmer, Amélie Servettaz, Chloé Stavris, Sébastien Trouillier

**Affiliations:** 1 Internal Medicine, Reference Center for Lysosomal Diseases (CRML), GH Diaconesses Croix Saint-Simon, Paris, France; 2 Reference Center for Inherited Metabolic Diseases, Hospices Civils de Lyon, Bron, France; 3 Laboratory Gillet-Mérieux, Centre de Biologie Et de Pathologie Est, INSERM U820, Hospices Civils de Lyon, Bron, France; 4 Biochemical and Molecular Biology Department, Centre de Biologie Et de Pathologie Est, Hospices Civils de Lyon, Bron, France; 5 Paediatric Neurology, Reference Center for Inherited Metabolic Diseases, CHU La Timone Enfants, Marseille, France; 6 Endocrinology, Diabetology, Metabolism Department, Reference Centre for Inherited Metabolic Diseases, Lille University Hospital, Lille, France; 7 Internal Medicine and Clinical Immunology, Competence Centre for Inherited Metabolic Diseases, Angers University Hospital, Angers, France; 8 Pediatric Neurology, Reference Center for Lysosomal Diseases, Armand Trousseau-La Roche Guyon Hospital, Assistance Publique-Hôpitaux de Paris, Fédération Hospitalo-Universitaire, Sorbonne-Université, I2-D2 Paris, France; 9 Reference Center for Lysosomal Diseases, Beaujon Hospital, Assistance Publique Hôpitaux de Paris Nord, Université Paris Cité, Paris, France; 10 Reference Center for Rare Pulmonary Diseases, Avicenne Hospital, Université Sorbonne Paris Nord, INSERM U1272, Assistance Publique-Hôpitaux de Paris, PneumologyBobigny, France; 11 Medical Genetics Unit, University Hospital of Bordeaux, INSERM U1211, Bordeaux, France; 12 Cancer Research Center of Toulouse (CRCT) and Clinical Biochemistry Laboratory, Reference Center for Inherited Metabolic Diseases, INSERM UMR1037 Paul Sabatier University Federative Institute of Biology, CHU Toulouse, Toulouse, France; 13 Sanofi, Gentilly, France; 14 Sanofi, Reading, UK; 15 Sanofi, São Paulo, Brazil; 16 Reference Center for Inherited Metabolic Diseases, Hôpital Necker Enfants Malades, Paris, 75015 France

**Keywords:** Acid sphingomyelinase deficiency (ASMD), Niemann–Pick disease, Mortality, Survival, Niemann–Pick disease type B, Standardised mortality ratio, France

## Abstract

**Background:**

Acid sphingomyelinase deficiency (ASMD) or Niemann–Pick disease types A, A/B, and B is a progressive, life-limiting, autosomal recessive disorder caused by sphingomyelin phosphodiesterase 1 (*SMPD1*) gene mutations. There is a need to increase the understanding of morbidity and mortality across children to adults diagnosed with ASMD.

**Methods:**

This observational retrospective survey analysed medical records of patients with ASMD with retrievable data from 27 hospitals in France, diagnosed/followed up between 1^st^ January 1990 and 31^st^ December 2020. Eligible records were abstracted to collect demographic, medical/developmental history, and mortality data. Survival outcomes were estimated from birth until death using Kaplan–Meier survival analyses; standardised mortality ratio (SMR) was also explored.

**Results:**

A total of 118 medical records of patients with ASMD (type B [*n* = 94], type A [*n* = 15], and type A/B [*n* = 9]) were assessed. The majority of patients were males (63.6%); the median [range] age at diagnosis was 8.0 [1.0–18.0] months (type A), 1.0 [0–3] year (type A/B), and 5.5 [0–73] years (type B). Overall, 30 patients were deceased at the study completion date; the median [range] age at death for patients with ASMD type A (*n* = 14) was 1 [0–3.6] year, type A/B (*n* = 6) was 8.5 [3.0–30.9] years, and type B (*n* = 10) was 57.6 [3.4–74.1] years. The median [95% confidence interval (CI)] survival age from birth in patients with ASMD type A and type A/B was 2.0 [1.8–2.7] years and 11.4 [5.5–18.5] years, respectively. Survival analysis in ASMD type B was explored using SMR [95% CI] analysis (3.5 [1.6–5.9]), which showed that age-specific deaths in the ASMD type B population were 3.5 times more frequent than those in the general French population. The causes of death were mostly severe progressive neurodegeneration (type A: 16.7%), cancer (type B: 16.7%), or unspecified (across groups: 33.3%).

**Conclusions:**

This study illustrated a substantial burden of illness with high mortality rates in patients with ASMD, including adults with ASMD type B, in France.

**Supplementary Information:**

The online version contains supplementary material available at 10.1186/s13023-024-03234-6.

## Background

Acid sphingomyelinase deficiency (ASMD, historically known as Niemann–Pick disease types A, A/B, and B) is a rare, autosomal recessive, lysosomal storage disease. It is caused by biallelic pathogenic variants in the sphingomyelin phosphodiesterase 1 (*SMPD1*) gene, which encodes the lysosomal enzyme acid sphingomyelinase (ASM; EC 3.1.4.12) [1–3]. Consequently, the ASM enzyme activity is reduced, and the corresponding substrates, primarily sphingomyelin, accumulate intracellularly [2–4]. The disease is pan-ethnic, with an estimated birth prevalence of 0.4–0.6 cases per 100,000 newborns [5–8]. As with other rare diseases, ASMD remains underdiagnosed in France, where the birth prevalence of ASMD type B has been estimated to be 1 per 230,000 births [9].

Patients with ASMD present with a wide clinical spectrum due to different *SMPD1* variants, variable residual ASM activity, and other genetic/epigenetic factors [10–12]. The clinical forms of ASMD are a continuum, as observed in Gaucher disease. The disease phenotypes have been broadly divided into three groups: type A (infantile neurovisceral), which is rapidly progressive and fatal in early infancy [4, 11, 13]; type B (chronic visceral), with no or little neurological manifestations [11, 12, 14]; and type A/B (intermediate chronic neurovisceral) [15–17].

In patients with ASMD type A, hepatosplenomegaly is the most common early symptom observed at 3 months of age or earlier, followed by the stagnation of psychomotor development before 12 months of age and relentless neurological degeneration [11, 13]. Most children with ASMD type A die before the third year of life [11, 14]. ASMD type B has a broad spectrum of disease severity. The clinical manifestations include hepatosplenomegaly, interstitial lung disease, thrombocytopenia with/without bleeding, delayed growth and puberty, dyslipidaemia, and osteoporosis/osteopenia [10, 18]. Respiratory and liver diseases are the leading causes of death in patients with ASMD type B [19]. In the more recently described ASMD type A/B, a variable degree of neurological impairment has also been reported; in some cases, learning difficulties or mild neuropathy were observed, while in other cases, the impairment was severe [10, 18, 20]. Most patients with ASMD type A/B die prematurely due to a combination of visceral and neurologic complications [16, 17].

The diagnosis of ASMD is often challenging and delayed due to overlapping symptoms with other chronic conditions. The diagnosis is based on reduced or undetectable ASM enzyme activity [10]. Olipudase alfa is an enzyme replacement therapy approved for treating non-central nervous system manifestations of ASMD in paediatric and adult patients across >35 countries, including Japan, the European Union, and the United States [21, 22]. Olipudase alfa was found to be well-tolerated for up to 2 years of treatment in adults and children diagnosed with ASMD type B or type A/B (NCT02004704) [23–26].

Given the rarity and heterogeneity of ASMD, robust and updated survival estimates to understand the impacts of the disease in its chronic forms are limited. Two studies have been conducted in the United States on survival analysis in patients with ASMD [27, 28]. McGovern et al. first analysed the morbidity and mortality of 103 patients followed up between 1992 and 2012, and reported 18 deaths with a median [range] age at death of 17.0 [2–72] years [27]. A recent report on 110 patients, covering the period 1990–2021, showed that the median age at death for patients with chronic ASMD was 21.3 years, and their life expectancy was around half of that in the general United States population, demonstrating substantial life shortening impact of this disease [28]. An international 11-year natural history study also described the spectrum of disease manifestations and disease-related morbidity and mortality in 59 patients, including seven patients from France [29]. There is a need to expand the knowledge of morbidity and mortality in patients with ASMD using retrospective studies with real-world data from different countries.

This retrospective study aimed to evaluate the impact of the disease on the survival of patients with ASMD in France.

## Methods

### Study design and participants

This multicentre retrospective chart review study was conducted in France to estimate the survival probability of paediatric, adolescent, and adult patients with ASMD.

To minimise selection bias and avoid restricted inclusion of patients already known to the French expert centres, referral hospitals for patients with ASMD diagnosed between 1990 and 2020 (in two diagnostic reference laboratories) were also identified. The physicians who had followed at least one patient with ASMD in France during the specified period were contacted.

All patients (surviving or deceased) diagnosed with ASMD and/or followed up in France, with retrievable information from the medical records between 1^st^ January 1990 and 31^st^ December 2020 were included in the study. Information was collected from the local treating physician. All patients had a proven ASMD diagnosis (deficient ASM activity and/or biallelic pathogenic *SMPD1* variants). Patients with no retrievable information from medical records or those who did not provide consent for data collection were excluded from the study. Duplicate data (records of patients followed in several centres or female patients with another name after marriage) were identified and consolidated. This study included a few patients from previously published studies or case reports [9, 30–34].

The primary objective was to evaluate the mortality in patients with ASMD type B during childhood, adolescence, and adulthood. The secondary objectives were to analyse the characteristics of patients with ASMD type A, type B, or type A/B and survival probability in ASMD type A or type A/B.

This study was conducted in accordance with the Declaration of Helsinki and all subsequent amendments, the guidelines for Good Epidemiology Practice, Good Pharmacoepidemiology Practices issued by the International Society for Pharmacoepidemiology, all national laws and regulations of France (where the study was performed), and any applicable guidelines. The necessary regulatory submission (Ethics Committee) was performed in accordance with local regulations, including local data protection regulations. A written and signed informed consent was obtained from alive participants or their legal representatives. The study was approved by the Health Data Hub on 9^th^ March, 2021.

### Data collection

Data were extracted retrospectively from patient’s medical records during the observation period, defined as the date of first retrievable information from the medical record until entry in this chart review. The study initiation date was the date of inclusion of the first patient with ASMD, and the study completion date was the date of inclusion of the last patient (study cut-off date), with an approximate extraction period of 12 months. The data on demographic characteristics, medical history (including age at first symptom, at diagnosis, and at last follow-up/death), past surgical history of splenectomy (and type of splenectomy), developmental history, survival status, and causes of death were retrieved from included patients. The physicians classified the patients based on the type of ASMD, using information retrieved from their medical records at the time of inclusion.

### Statistical analyses

The data were summarised using descriptive statistics. The proportion of missing data was indicated where appropriate. Continuous variables were presented as mean and standard deviation or median and range at each longitudinal time point, and categorical variables were expressed as number and percentage.

The survival outcomes for patients with ASMD type A, type A/B, or type B were estimated using Kaplan–Meier (KM) analysis. Overall survival (OS) was defined as the time between the birth date and the death date or censoring. Further, median OS with two-sided 95% confidence intervals (CIs) was estimated. The data for patients who were alive at the end of the study date or lost to follow-up were censored at the last date they were known to be alive.

A standardised mortality ratio (SMR) analysis was estimated *post hoc* to calculate the survival probability of patients with ASMD type B and chronic ASMD (type B and type A/B). The SMR was calculated using the formula:

$$\text{SMR}=\frac{\text{The actual number of deaths}}{\text{The expected number of deaths}}$$

The 95% CI around the SMR estimate was also calculated. The expected number of deaths was based on the age-specific mortality rates in the general population and applied to the number at risk in the ASMD database. Mortality data for the general population were obtained from published life tables for France [35]. The survival probabilities for patients with ASMD were estimated using the calculated SMR applied to the mortality data of the general population.

The SMR computation was performed using Microsoft Excel (version 2208). All other analyses were conducted using SAS statistics software version 9.4 (SAS Institute Inc., Cary, NC, USA). The proportion of missing data was summarised where appropriate.

## Results

### Patient characteristics

A total of 29 investigators from 27 participating French hospitals were involved in a 12-month enrolment period. The two most common participating specialities/departments were Internal Medicine (15 sites) and Paediatrics (7 sites); others were Pneumology (4 sites), Haematology (1 site), Endocrinology (1 site), and Medical Genetics (1 site). The median [range] number of patients included per site was 2.0 [1.0–28.0], and the main recruiters were two sites of Internal Medicine and Paediatrics, both of which were referral centres for rare diseases.

Following the retrospective chart extraction, the patients were again followed up over a median [range] period of 4.8 [0–10.0] months; however, the survival status did not change between the chart extraction period and the final study cut-off date.

All 118 screened patients with ASMD were included in the study. The majority of them were identified with ASMD type B (*n* = 94; 79.7%), followed by ASMD type A (*n* = 15; 12.7%) and ASMD type A/B (*n* = 9; 7.6%) (Table 1). There was a male predominance in the overall population (63.6%), primarily in the ASMD type B population (68.1%).

**Table 1:** Patient baseline characteristics

Parameter	Overall	ASMD type A	ASMD type A/B	ASMD type B
**Total population [*n*]**	118	15	9	94
**Sex [*n* (%)]**				
Male	75 (63.6)	7 (46.7)	4 (44.4)	64 (68.1)
Female	43 (36.4)	8 (53.3)	5 (55.6)	30 (31.9)
**Survival status of patients [*n* (%)]**				
Alive	88 (74.6)	1^a^ (6.7)	3 (33.3)	84 (89.4)
Dead	30 (25.4)	14 (93.3)	6 (66.7)	10 (10.6)
**Age at first symptom onset**				
Number^b^	95	13	8	74
Mean (SD)	12.9 (18.0) years	7.2 (4.7) months	1.0 (0.9) year	16.4 (19.0) years
Median [range]^c^	3.0 [0–61.0] years	6.0 [3.0–18.0] months	1.0 [0–3.0] year	5.0 [0–61.0] years
**Age at diagnosis**				
Number^b^	115	14	9	92
Mean (SD)	15.0 (20.3) years	7.6 (4.6) months	1.4 (1.1) years	18.6 (21.2) years
Median [range]^c^	3.0 [0–73.0] years	8.0 [1.0–18.0] months	1.0 [0–3.0] year	5.5 [0–73.0] years
**Age at last follow-up for alive patients**^**d**^** (years)**				
Number^b^	88	1^a^	3	84
Mean (SD)	33.5 (19.5)	1.0	6.3 (4.9)	34.9 (18.9)
Median [range]	35.5 [1.0–74.0]	1.0	4.0 [3.0–12.0]	36.5 [1.0–74.0]
**Age at death**^**e**^** (years)**				
Number	30	14	6	10
Mean (SD)	15.0 (24.0)	1.6 (1.3)	12.3 (9.8)	47.0 (22.8)
Median [range]^c^	3 [0–74.0]	1 [0–3.6]^f^	8.5 [3.0–30.9]	57.6 [3.4–74.1]

*ASMD* Acid sphingomyelinase deficiency, *n* Number of patients, *SD* Standard deviation

^a^The patient died at the age of 2 years 7 months in December, 2022 (2 years after the cut-off date)

^b^Some patients were excluded from the analysis due to the lack of information

^c^Age between 0 and 1 year has been represented as “0” in the descriptive statistics

^d^All alive patients at the cut-off date

^e^Age at the death of deceased patients

^f^Two patients with ASMD type A died at 3 and 9 months, respectively

Patients with ASMD type A experienced early symptom onset (median [range] age: 6.0 [3.0–18.0] months) and diagnosis (median [range] age: 8.0 [1.0–18.0] months) with median [range] age at death being 1 [0–3.6] year(s). Patients with ASMD type A/B had first symptom onset in early childhood (median [range] age: 1.0 [0–3] year), with median [range] age being 8.5 [3.0–30.9] years at death (Table 1). In the ASMD type B cohort, the age at first symptom onset and diagnosis varied widely, with median [range] age being 5.0 [0–61.0] years and 5.5 [0–73.0] years, respectively (Table 1).

The majority (60.9%) of patients (*n* = 92) with ASMD type B were <10 years old at diagnosis, a few (2.2%) were in the range of 10–18 years, and 37.0% were adults (>18 years) (Supplementary Table 1). The median [range] age of diagnosis for the adults was 9 [0–71] years.

### Mortality characteristics

At the cut-off date, a total of 30/118 (25.4%) patients had died. Patients with ASMD type A had the highest mortality rate (14/15, 93.3%); the only patient still alive at the cut-off date died 2 years later (December, 2022) at the age of 2 years 7 months. The mortality rate of patients with chronic ASMD type A/B was also high (6/9, 66.7%). For patients with ASMD type B, a mortality rate of 10.6% (10/94) was observed (Table 2).

**Table 2:** Survival status of patients with ASMD

Parameter	Overall (*N* = 118)	ASMD type A (*n* = 15)	ASMD type A/B (*n* = 9)	ASMD type B (*n* = 94)
**Survival status at the cut-off date [*n* (%)]**				
Deceased patients	30 (25.4)	14 (93.3)	6 (66.7)	10 (10.6)
**Causes of death [*n* (%)]**				
Number	30	14	6	10
Cancer	5 (16.7)	0	0	5 (50.0)
Severe progressive neurodegeneration	5 (16.7)	5 (35.7)	0	0
Hepatic disease	2 (6.7)	2 (14.3)	0	0
Respiratory insufficiency	2 (6.7)	2 (14.3)	0	0
Bleeding	1 (3.3)	0	1 (16.7)	0
Cardiovascular	1 (3.3)	0	0	1 (10.0)
Complication following liver transplantation	1 (3.3)	0	0	1 (10.0)
Hepatic disease with complications after transplantation	1 (3.3)	0	0	1 (10.0)
Hepatic disease, respiratory insufficiency, and pneumonia	1 (3.3)	0	1 (16.7)	0
Sudden faintness with bradycardia without initial desaturation	1 (3.3)	0	0	1 (10.0)
Unknown	10 (33.3)	5 (35.7)	4 (66.7)	1 (10.0)
**Age at the first symptom onset of deceased patients (years**^**a**^**)**				
Number^b^	22	12	6	4
Mean (SD)	6.1 (15.8)	7 (5.0)	1.2 (1.0)	31.5 (26.3)
Median [range]^c^	0 [0–57]	5.0 [2–17]	1.0 [0–3]	34.5 [0–57]
**Age at diagnosis of deceased patients (years**^**a**^**)**				
Number^b^	27	13	6	8
Mean (SD)	9.7 (20.3)	7 (5.0)	1.5 (1.2)	31.3 (27.8)
Median [range]^c^	1.0 [0–73]	7.0 [1–18]	1.0 [0–3]	36.5 [0–73]

*ASMD* Acid sphingomyelinase deficiency, *n* Number of patients, *SD* Standard deviation

^a^Age of patients with ASMD type A is provided in months

^b^Some patients were excluded from the analysis due to missing information

^c^Age between 0 and 1 year was represented as ‘0’ in the descriptive statistics

**Fig. 1:**
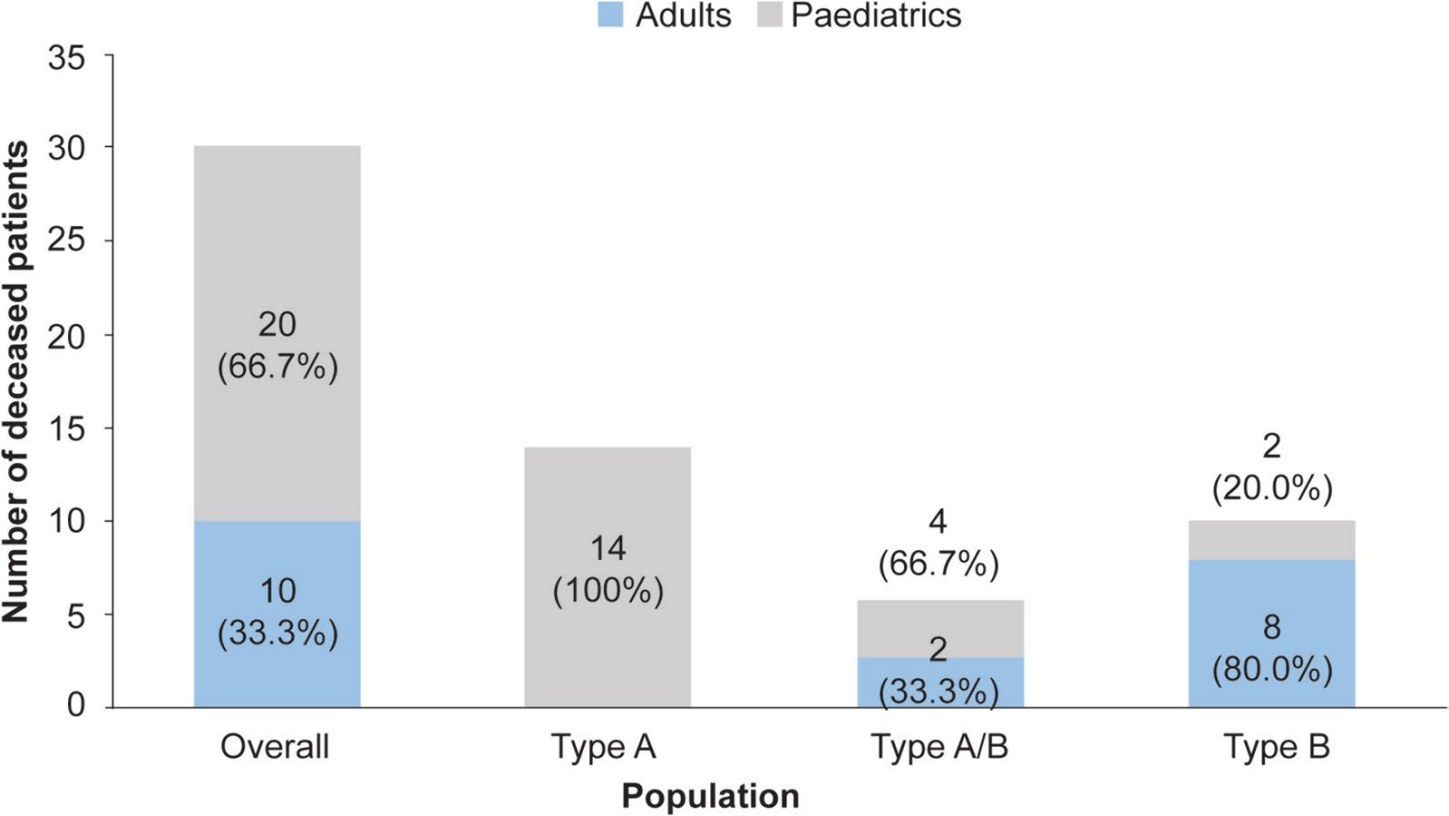
Distribution of deceased patients with ASMD based on phenotype and age category at death. The numbers on/around the bars represent the number of deceased patients, *n* (%). ASMD, acid sphingomyelinase deficiency

At death, all patients with ASMD type A (*n* = 14) were aged ≤3.6 years, whereas those with type A/B included both children (<12 years, *n* = 4) and adults (≥18 years, *n* = 2). Deceased patients with ASMD type B were mostly adults (*n* = 8) at death (Fig. 1); only two patients died during childhood (3.0 and 9.0 years). Interestingly, the median age at diagnosis for the deceased patients with ASMD type B was substantially higher (36.5 [0–73] years) (Table 2) than that for the overall type B cohort (5.5 [0–73.0] years) (Table 1).

The causes of death were unknown for most patients with ASMD (33.3%). The most common cause of death in patients with ASMD type A was severe progressive neurodegeneration (5/14, 35.7%). Adult patients with ASMD type B died due to cancer (5/10, 50%), including lung cancer (*n* = 2), bladder cancer (*n* = 1), head and neck cancer (*n* = 1), and one case of cancer of an unknown origin (Table 2).

Six (5.1%) patients with ASMD (all with type B) had undergone splenectomy; among them, three were reported dead before inclusion in the study (two in childhood and one in adulthood).

### Overall survival in patients with ASMD

**Fig. 2:**
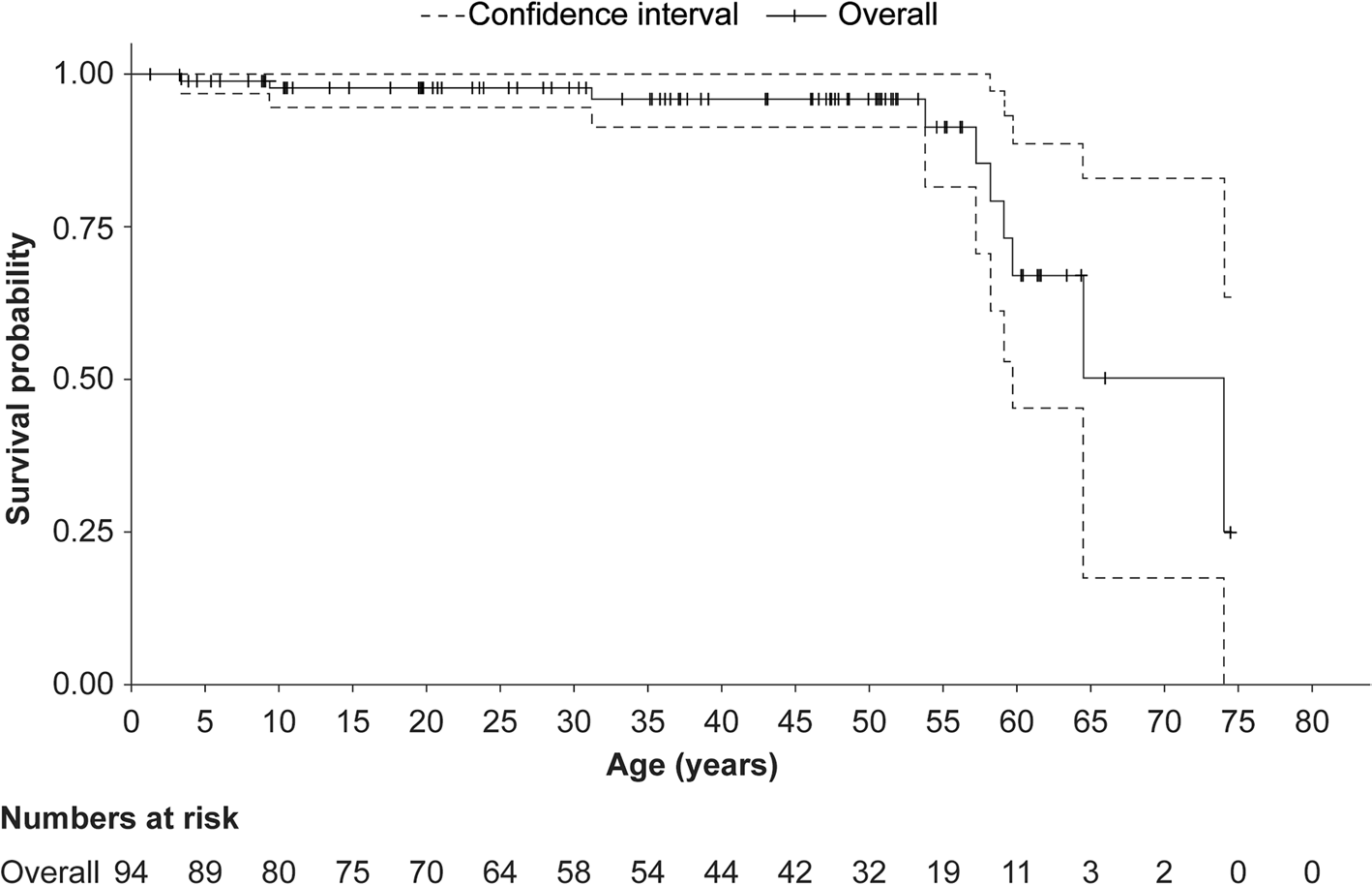
Kaplan–Meier survival curve since birth for patients with ASMD type B. ASMD, acid sphingomyelinase deficiency

The 95% CI of the KM survival curve since birth for patients with ASMD type B was not obtainable due to the low number of patients at risk, especially after the age of 60.0 years, leading to uncertainty in reporting the median OS age (Fig. 2). There was heavy censoring of the ASMD type B population, with only 42 (45.0%) patients alive at the age of 40.0 years and left at risk (Supplementary Fig. 1).

**Fig. 3:**
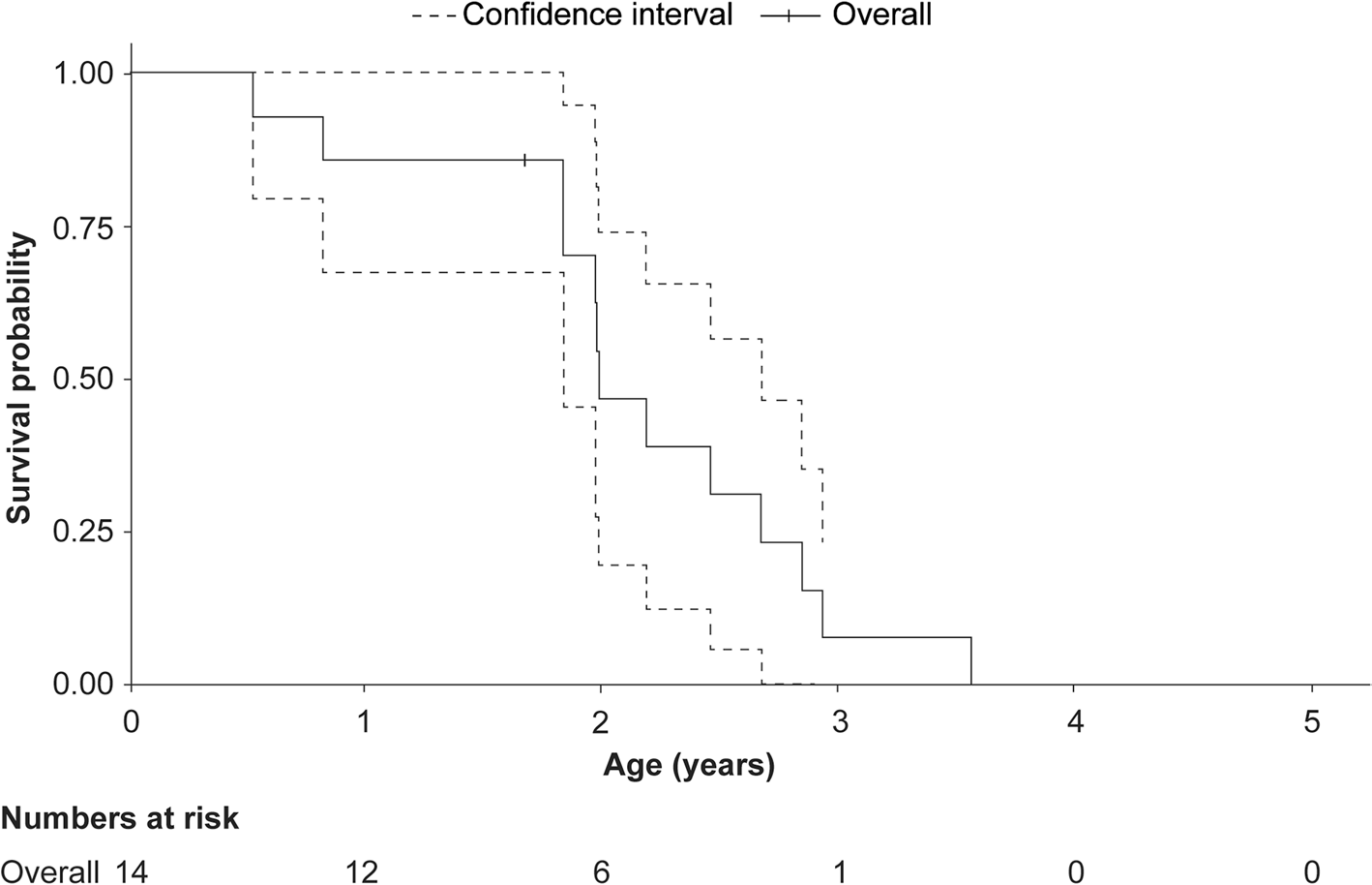
Kaplan–Meier survival curve since birth for patients with ASMD type A. ASMD, acid sphingomyelinase deficiency

**Fig. 4:**
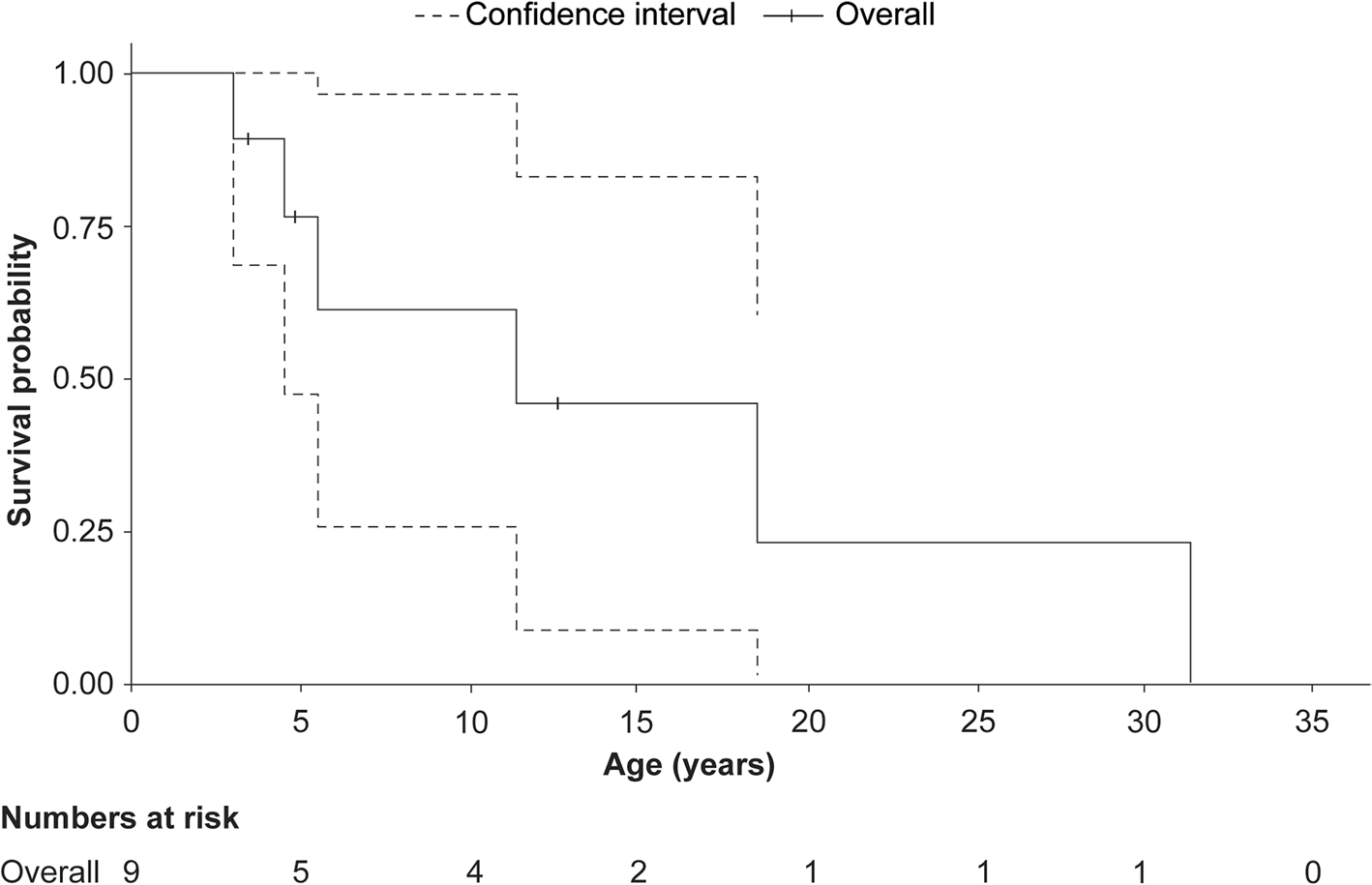
Kaplan–Meier survival curve for patients with ASMD type A/B. ASMD, acid sphingomyelinase deficiency

Furthermore, KM curve analyses for patients with ASMD type A yielded a median [95% CI] OS age of 2.0 [1.8–2.7] years (Fig. 3) as the 95% CI values were obtainable. The 95% CI for the KM survival curve since birth for patients with ASMD type A/B was wide due to small sample size (*n* = 9) but was obtainable, and the median OS age of 11.4 [5.5–18.5] years could be reported (Fig. 4).

**Fig. 5:**
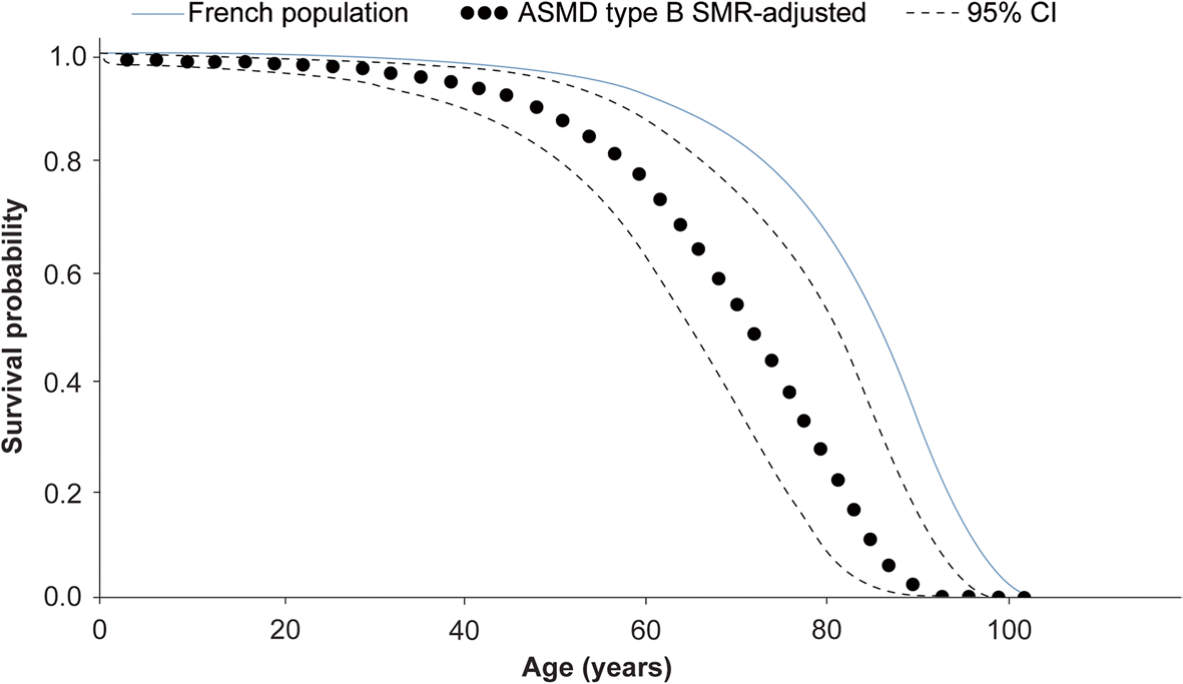
Survival probabilities for patients with ASMD type B compared with the general French population. ASMD, acid sphingomyelinase deficiency; CI, confidence interval; SMR, standardised mortality ratio

In addition to the KM estimation, an SMR was additionally calculated for the ASMD type B population to compare their mortality risk with that of the general French population for understanding the impact of the disease. The estimated SMR (95% CI) of 3.5 (1.6–5.9) was applied to the mortality rates of the French population as a multiplier to calculate adjusted survival probabilities (Fig. 5). This result indicated that the ASMD population in France had 3.5-times more deaths than the age-specific mortality rates in the general population.

Further, the SMR [95% CI] was calculated for the overall population with ASMD type B and type A/B and was estimated to be 5.5 [3.1–8.5], indicating a high severity of ASMD type A/B in this French population.

## Discussion

Reports on survival analysis and causes of death in patients with ASMD in France or other European countries are lacking. Herein, using medical records collected from French hospitals, the survival of children, adolescents, and adult patients was analysed based on their ASMD phenotype, ranging from rapidly progressing infantile neurovisceral disease to slow chronic visceral disease.

The significant number of participating sites, including expert centres in metabolic/lysosomal diseases, their geographical distribution, and the number of included patients, ensured a fair representation of the comprehensive population of patients with ASMD, particularly for type B, in France. The well-structured organisation of expert centres for metabolic/lysosomal diseases in this country allowed easy data extraction.

The included patients were ascribed to one of the three classical ASMD phenotypes based on their symptoms and disease course. The classification into ASMD type A, type A/B, or type B was generally clear, and only a few patients were at the borderline of type A and type A/B phenotypes. The study population primarily included patients with ASMD type B (79.7%). This percentage was slightly higher than that reported for a large cohort of patients diagnosed in France (73%) during the overall period of 1974–2021 [36]; this difference can largely be explained by a proportionally higher number of adult patients with ASMD type B diagnosed in the last 10 years.

In this study population, the proportion of male patients was considerably higher than that of the female population in the ASMD type B cohort (68.1% vs 31.9%). Interestingly, a similar trend (71.0% vs 29%) was observed in a recent United States cohort [28]. In this study, the majority of patients with ASMD type B showed the first symptom onset (63.5%) and diagnosis (63.1%) of the disease at paediatric age (<18 years) (median age, 3.0 years at both time points). However, 36.5% of patients with ASMD type B (median [range] age: 37.0 [20–61] years) developed their first symptoms and 37% (median [range] age: 42.5 [20–73] years) were diagnosed with the disease at ≥18 years. In the recently published study in ASMD type B cohort in the United States, the global age at onset or diagnosis was 3.3 and 3.7 years, respectively, with 80% of the patients diagnosed in paediatric age (<18 years), indicating a significantly global younger age of the patients [28]; this was also a characteristic of the cohort reported by McGovern et al. earlier [27].

In our study, for patients with ASMD type A or type A/B, 100% of the patients showed their first symptom or diagnosis at paediatric age.

A global mortality rate of 25.4% was observed for all patients with ASMD. In patients with ASMD type B, the mortality rate was 10.6%, with 80% of deaths recorded in adults (median age at death: 58.5 years). The majority of patients with ASMD type B (*n* = 7) who died in adulthood (>40 years) had been diagnosed after the age of 10.0. Only two deaths occurring in childhood (age at death: 3.0 and 9.0 years, respectively) were reported. Previously, McGovern et al*.* reported a global mortality rate of 17.5% in a cohort of 103 patients categorised as type B in the United States between 1992 and 2012, with a high mortality rate (19%) in the paediatric (<21 years) subcohort (59% of patients) [27]. However, their population also included 13 patients with neurologic manifestations, indicating enrolment of some patients with ASMD type A/B with poor prognoses. In a recently published United States study [28], the median age at death for the ASMD type B subpopulation was 6.7 years, with a high mortality rate of 33.3%. In comparison with both United States studies, the mortality rate found in the present cohort thus appears rather low. Moreover, in the study by Pulikottil-Jacob et al., only 26% of the deceased patients with ASMD type B were adults (>18 years) [28] compared with 80% in the French cohort of this study. We further observed that a high proportion of deceased patients with ASMD type B had been diagnosed or followed up in mid- and late-adulthood. Of note, in this retrospective study, only limited data could be retrieved to conclude regarding the age at first symptoms, which might have occurred much earlier, especially for the older patients. The underdiagnosis of late-onset ASMD in the United States could partly explain differences between cohorts. The prognosis of ASMD type B in France may also differ from that in the United States due to a dissimilar distribution of genetic variants [36]. The common *SMPD1* variant p.R610del, exclusively observed in patients with ASMD type B [37, 38], has been reported to be more frequent in patients from Spanish and French extraction and is highly prevalent in patients originating from North Africa [39]. In a cohort of 115 fully genotyped unrelated patients with ASMD type B followed up in French hospitals during the period 1974–2021, p.R610del constituted globally 54% of the alleles, in part due to the significant participation of patients with North African roots [36]; an even higher frequency of p.R610del was also found in a previous French clinical study in 28 adult patients [9]. This allele is generally considered associated with an attenuated phenotype [29]. In the present study, *SMPD1* gene mutations were not analysed and ethnicity was not recorded, preventing a specific discussion on genotype–phenotype correlations in this cohort; a high representation of the p.R610del allele is nevertheless highly probable.

The most common causes of death reported in patients with ASMD type B are pneumonia/respiratory failure, acute liver failure, bleeding complications and complications from bone marrow transplants, multi-organ failure, heart failure, and liver cancer [14, 19]. In this study, the causes of death were slightly different from previously reported data. Half of the deaths in patients with ASMD type B were due to cancer, including cancers in the lung, bladder, and head and neck, as well as a case with an unknown cancer. Previously, Mauhin et al. have reported a high prevalence of cancer (16.1%), including breast cancer, lung cancer, thyroid cancer, and bladder cancer in a French population of 31 patients with chronic ASMD [30]. These patients were also included in the present study. Previously, an analysis of the causes of death of 52 patients with chronic ASMD (age: 43–65 years) had shown a 9.6% prevalence of cancer, including liver cancers, multiple myeloma, chondrosarcoma, and one unknown type [19]. A study by McGovern et al. in patients with chronic ASMD reported one patient with liver cancer [29]. Interestingly, two paediatric deaths in the present study were due to complications following liver transplantation or sudden faintness with bradycardia without initial desaturation.

For patients with ASMD type A, a mortality rate of 93.3% was found at the cut-off date, with median OS since birth of 24 months; one patient who was reported alive at the cut-off date died 2 years later at the age of 2 years 7 months. These findings are in good agreement with the study by McGovern et al., which reported a median time of 21 months from diagnosis to death, with all patients succumbing in ≤3 years [11]. The high severity of the disease in patients with ASMD type A is due to multisystem manifestations associated with rapidly progressive neurologic involvement [40]. In this French ASMD type A cohort, severe progressive neurodegeneration was the cause of death in 35.7% of the cases; hepatic disease or respiratory insufficiency in 28.6%; and in 35.7%, the cause was unspecified.

In this study, patients with ASMD type A/B had a high mortality rate of 66.3%, i.e. much higher than the 33% found in the recent United States study [28]; these patients were characterised by a slower progression of neurologic symptoms and longer survival than those with ASMD type A [15–17, 19]. Neurodegeneration (23.1%), respiratory disease (23.1%), and liver disease (19.2%) are reported as the major causes of death in patients with ASMD type A/B [19]. Unfortunately, in the present study, the causes of death in patients with ASMD type A/B were mostly unknown (66.7%). One death resulted from bleeding, and another was due to the combined effects of liver disease, respiratory insufficiency, and pneumonia. The median OS [CI] age of patients with ASMD type A/B since birth was 11.4 [5.5–18.5] years, indicating a high severity of the disease; however, this data is uncertain and representative of a very small sample size.

For the ASMD type B population, median survival could not be estimated due to the low number of patients at risk. Apart from survival analyses using KM curves, alternative methods of evaluating OS may be helpful for precise mortality estimates because of the high proportion of censoring during these studies [29] and in rare conditions, such as ASMD. The calculated overall SMR was 3.5 in ASMD type B, indicating that there were approximately 3.5-times more deaths in the ASMD type B population compared to the age-specific mortality rates in the general French population. For the chronic ASMD population (ASMD type B and type A/B), the mortality rate was 5.5-times more than that for the non-affected population.

Severe splenomegaly and splenectomy are risk factors for death, with an increased risk of early mortality in ASMD [19, 29]. Given its retrospective nature, it was challenging to determine the impact of splenectomy on the progression of the disease in the present study. Splenectomy was noted only in six patients with ASMD type B, and three splectomised patients were deceased, indicating that 33.3% (3/10) of the deceased patients with ASMD type B had a history of splenectomy.

This study on the survival status of 118 patients in France highlights the need for early diagnosis, appropriate management, and regular follow-up to reduce the risk of complications and mortality.

The following limitations, however, should be considered while interpreting the study findings. One of the major limitations of the study was that the data were collected retrospectively from available medical records. The analysed population is representative but not comprehensive because 15 centres with potential patients did not participate for the following reasons: agreement not finalised (*n* = 5), centres not participating in clinical trials (*n* = 2) or not interested (*n* = 3), and unsuccessful selection (*n* = 5). However, the health structure for rare diseases in France organised in specialised centres, together with the need for a multidisciplinary follow-up of patients with ASMD, greatly reduced the number of potential non-included patients. Data on *SMPD1* gene variants were not collected, resulting in a lack of understanding on the genotype–phenotype correlations in ASMD. Moreover, this study estimated the mortality of patients with ASMD, but no information on morbidity and quality of life of alive patients was recorded. Similarly, symptoms and medical history data were not collected to validate the disease characterisation. Despite the wide survey period, this study did not include any patient with the year of death before 2005, did not identify any deceased patient among those diagnosed before 1980, and did not identify any paediatric death among those diagnosed before 1994. The possible contributing factors were patients followed by clinicians other than the investigators, a lack of a French ASMD registry, impossibility of exploring medical records that were not computerised in those years, and the investigator’s oversight of old facts. Furthermore, a peak in ASMD diagnoses was observed in France after 2010. This could be explained by the olipudase alfa clinical development and the recent structuring of rare disease networks in France (since 2004) that might have increased the awareness of this rare disease in the healthcare community and reduced diagnostic wandering. Finally, the OS for patients with ASMD type B was based on a limited number of events (*n* = 10) during the observation time. Heavy censoring was noted in these patients by the age of 40 years, leaving only a few patients at risk. Hence, there was uncertainty in estimating the median OS. Although this study used SMR as an alternative approach, its calculation relied on the available information from the patient charts and assumed a constant increase in mortality risk across ages. It might thus not represent the precise shape of the survival function of patients with ASMD.

## Conclusions

This is the first study to examine the survival of patients with ASMD in France over the last 30 years. While ASMD type A is fatal in early ages, ASMD type A/B has an early onset and is associated with high mortality.

This study confirms that although ASMD type B is mostly a paediatric discovery, symptom onset and diagnosis in adult patients is common in France. The adult patients with ASMD type B who died exhibited a high median age of diagnosis similar to that observed in the French population with the p.Arg610del variant; however, genotype–phenotype correlation is warranted for confirmation. Cancer was the major cause of death in this population. The increased cancer rate must be considered for proper patient follow-up and needs further validation in future studies.

A multidisciplinary follow-up is necessary for therapeutic decision-making to reduce the substantial impact of ASMD.

### Supplementary Information

Supplementary Material 1.

## Data Availability

The datasets used and/or analysed during the study are available from the corresponding author upon reasonable request.

## Abbreviations

ASM: Acid sphingomyelinase
ASMD: Acid sphingomyelinase deficiency
CI: Confidence interval
KM: Kaplan–Meier
*n*: Number of patients
OS: Overall survival
SD: Standard deviation
*SMPD1*: Sphingomyelin phosphodiesterase 1
SMR: Standardised mortality ratio
