# Supplementary material for: Acid sphingomyelinase deficiency in France: a retrospective survival study
Source: Orphanet J Rare Dis. 2024 Aug 5;19:289. doi: 10.1186/s13023-024-03234-6 (PMC11301966; doi:10.1186/s13023-024-03234-6)
Supplement: Supplementary file 1 — Supplementary Material 1. [file 13023_2024_3234_MOESM1_ESM.docx]

**Supplementary Table 1** Age categories of all patients with ASMD at the first symptom onset and diagnosis

| **Parameter** | **Overall (*N* = 118)** | **ASMD type A (*n* = 15)** | **ASMD type A/B  (*n* = 9)** | **ASMD type B  (*n* = 94)** |
| --- | --- | --- | --- | --- |
| **Age categories (years) at first symptom onset [*n* (%)]** | | | | |
| Number^a^ | 97 | 15 | 8 | 74 |
| Children [<10] | 68 (70.1) | 15 (100.0) | 8 (100.0) | 45 (60.8) |
| Children [10 to <18] | 2 (2.1) | 0 | 0 | 2 (2.7) |
| Adults ≥18 | 27 (27.8) | 0 | 0 | 27 (36.5) |
| **Age categories (years) at diagnosis [*n* (%)]** | | | | |
| Number^a^ | 116 | 15 | 9 | 92 |
| Children [<10] | 80 (69.0) | 15 (100.0) | 9 (100.0) | 56 (60.9) |
| Children [10 to <18] | 2 (1.7) | 0 | 0 | 2 (2.2) |
| Adults ≥18 | 34 (29.3) | 0 | 0 | 34 (37.0) |

^a^Some patients were excluded from the analysis due to missing information.

ASMD, acid sphingomyelinase deficiency; *n*, number of patients


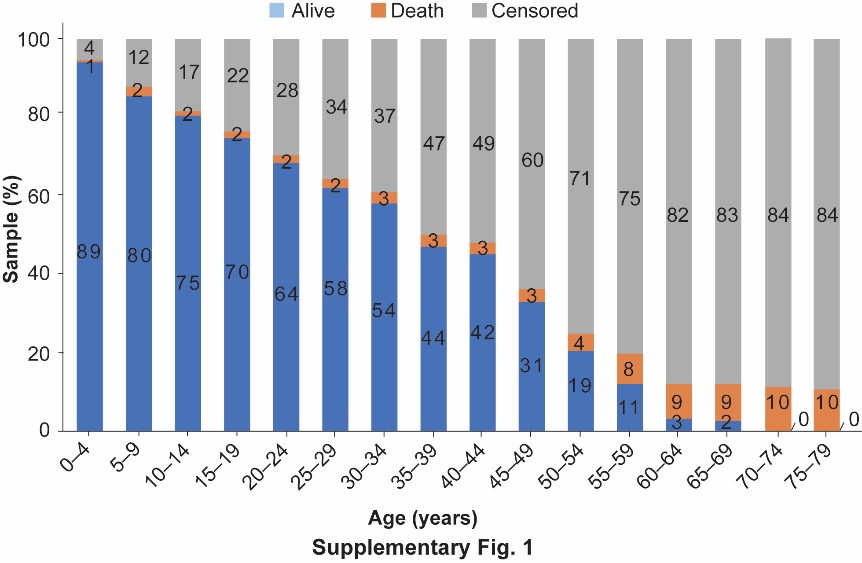


**Supplementary Fig. 1** Sample breakdown of data used for an event occurring or censoring. The numbers on the bars represent the actual number of patients with ASMD type B (n = 94).

ASMD, acid sphingomyelinase deficiency; n, number of patients
